# Antioxidant Mechanisms Underlying the Gastroprotective Effect of Menthofuran on Experimentally Induced Gastric Lesions in Rodents

**DOI:** 10.1155/2023/9192494

**Published:** 2023-04-07

**Authors:** Naira Moura Alves, Paulo Humberto Moreira Nunes, Anderson Mendes Garcez, Manoela Carine Lima de Freitas, Irisdalva Sousa Oliveira, Francilene Vieira da Silva, Hélio de Barros Fernandes, Damião Pergentino de Sousa, Rita de Cássia Meneses Oliveira, Daniel Dias Rufino Arcanjo, Maria do Carmo de Carvalho e Martins

**Affiliations:** ^1^Medicinal Plants Research Center, Federal University of Piauí, Teresina, PI, Brazil; ^2^Department of Biophysics and Physiology, Federal University of Piauí, Teresina, PI, Brazil; ^3^Department of Pharmaceutical Sciences, Federal University of Paraíba, João Pessoa, PB, Brazil

## Abstract

Menthofuran is a monoterpene present in various essential oils derived from species from *Mentha* genus, and in Brazil, those species are widely used in treating gastrointestinal and respiratory disorders. Considering the wide pharmacological potential of monoterpenes, including their antioxidant activity, this study aimed to evaluate menthofuran-gastroprotective activity, as well as the involvement of antioxidant mechanisms in this effect. The acute toxicity was evaluated according to the fixed dose method. The antiulcerogenic activity was investigated by using experimental models of gastric ulcers induced by ethanol, indomethacin, and ischemia/reperfusion in rats. The antisecretory gastric activity, the catalase activity, and the gastric wall mucus were determined in pylorus ligated rats. Gastric wall nonprotein sulfhydryl (NPSH) group content, myeloperoxidase (MPO) activity, and malondialdehyde (MDA) content were evaluated in ethanol-induced the gastric ulcer model. Menthofuran (2 g/kg) presented low acute toxicity and showed gastroprotective activity against ethanol-, indomethacin-, and ischemia/reperfusion-induced ulcers. Moreover, menthofuran presented antisecretory activity, reduced the total acidity, and increased pH of gastric secretion. On the other hand, a decrease in mucus content of gastric wall without alteration of gastric juice volume and catalase activity was observed. Interestingly, menthofuran increased NPSH levels and reduced MDA levels and MPO activity. Gastroprotective effects of menthofuran appear to be mediated, at least in part, by the NOS pathway, endogenous prostaglandins, reduced gastric juice acidity, increased concentration of the NPSH groups, and reduced lipidic peroxidation. These findings support the menthofuran as an effective gastroprotective agent, as well as the marked participation of antioxidant mechanisms in this response.

## 1. Introduction

Gastric ulcer is a relevant health problem, since it occurs from 12 to 17% in developed countries [1]. This gastrointestinal disorder affects 10% of the population at some period of life [2]. Gastric ulcer is caused by an imbalance between the action of aggressive and defensive factors on the gastric mucosa [3]. Gastric mucosal damage can be induced by aggressive factors such as hydrochloric acid, pepsin, leukotrienes, free radicals, nonsteroidal anti-inflammatory drugs (NSAIDs), ischemia, dysmotility, ethanol, nicotine, and stress [4].

There are several effective drugs in the treatment of gastric and duodenal ulcers, among which are inhibitors of proton pump (omeprazole and lansoprazole) and antagonists of histamine H_2_ receptor (ranitidine and famotidine) that interfere with the secretion of acid, and antacids that neutralize acid secretion [5, 6]. Cytoprotective agents such as sucralfate and colloidal bismuth are also effective [5]. However, many of these drugs besides having excessive costs produce different adverse effects including hypersensitivity, arrhythmias, hematopoietic disorders, erectile dysfunction, and gynecomastia [7, 8].

Many medicinal plants extracts are used in folk medicine in Brazil to treat diverse types of digestive disorders [9]. Several different substances found in these plants have gastroprotective effects [10] and among the major classes of compounds related to this activity, there are the terpenes, triterpenes, flavonoids, alkaloids, glycosides, saponins, and polysaccharides [11]. Further studies are needed to identify low-cost natural products with antiulcer activity showing effectiveness and low toxicity [9], since natural products represent a promising and renewed therapeutic strategy in gastroprotection.

The monoterpenes are volatile basic constituents of aromatic essential oils, and they are part of a group of chemical compounds with great structural variety and different biological activities, such as antiulcer [12], antimicrobial [13], antispasmodic [14], and antinociceptive activities [15]. The menthofuran is a monoterpene found in various essential oils derived from species such as *Mentha piperita* L., of the family *Lamiaceae* (Labiatae). They are catalogued, around 25 species, from the genus *Mentha.* In Brazil, leaves and branches from those species are widely used in treating gastrointestinal disorders [16] and respiratory disorders [17, 18], as well as worm infesting [19, 20]. Moreover, there are popular allegations of antispasmodic, analgesic, anti-inflammatory, antifungal, and antiseptic activities related to these plants [21, 22].

Considering the wide pharmacological potential of monoterpenes, including their antioxidant activity, this study aimed to evaluate menthofuran-gastroprotective activity, as well as the involvement of antioxidant mechanisms in this effect.

## 2. Materials and Methods

### 2.1. Animals

Male Wistar rats (70–90 days old and 250–300 g) and male Swiss mice (60–70 days old and 30–40 g) were used for the study. Animals were provided by the Sectorial Vivarium of the Medicinal Plant Research Center of Federal University of Piaui. They were maintained in proper conditions, temperature of 25 ± 2°C, approximately 60% humidity, and 12 h light/dark cycles. The animals were provided with a rodent-pellet diet (LABINA 5002, EVIALIS do Brasil Nutrição Animal Ltda., Sao Paulo, Brazil) and water ad libitum. The animals were randomly assigned to different control and treatment groups. The experimental protocols were conducted with 7 animals/groups in accordance with the guidelines of the Brazilian Council of Animal Experimental Control and approved by the Ethics Committee for Animal Research at the Federal University of Piaui (Protocol N° 086/15).

### 2.2. Chemicals and Drugs

The following drugs and chemicals were used: absolute ethanol (Impex, Brazil), ketamine and xylazine (Syntec, Brazil), Alcian blue, carbenoxolone, dithio-bis-nitrobenzoic acid, ethylenedinitrilo-tetraacetic acid (EDTA), indomethacin, malondialdehyde tetrabutylammonium salt, menthofuran, N-acetylcysteine, ranitidine, sodium acetate, 2-thiobarbituric acid, trichloroacetic acid and Tween 80 (all from Sigma-Aldrich, St. Louis, USA), and sodium thiopental (Cristália, Brazil). The menthofuran was purchased from Sigma-Aldrich, St. Louis, USA.

All solutions of substances were prepared immediately before each experiment, using as a vehicle Tween 80 1% in 0.9% saline solution or distilled water. The menthofuran and drug concentrations were adjusted for treatment to yield a volume of 5 mL/kg for rats and 10 mL/kg for mice.

### 2.3. Evaluation of the Acute Oral Toxicity in Mice

This assay was performed according to the fixed dose method proposed by the Organization for Economic Cooperation and Development [23]. Male mice were randomized into groups of 7 animals and treated orally with single dose of vehicle (0.1% Tween 80, 5 mL/kg) or menthofuran (2 g/kg). The animals were observed for up to 8 hours on the first day of treatment and daily thereafter for 14 days. The following parameters were evaluated: alertness, sedation, ptosis, dyspnea, urination, diarrhea, convulsions, spontaneous motor activity, postural reflex, piloerection and response to touch (for 8 hours), and number of deaths (for 14 days).

### 2.4. Acute Gastric Ulcer Induced by Ethanol in Rats

The antiulcerogenic activity of menthofuran or carbenoxolone was investigated by using the acute ethanol-induced gastric ulcer in rats [24]. Male Wistar rats (7 animals/group) maintained under standard conditions as described above were fasted for 24 h and orally received vehicle (Tween 80 1%, 5 mL/kg), menthofuran (12.5, 25, 50. and 100 mg/kg), or carbenoxolone (250 mg/kg). One hour later, gastric lesions were induced by oral administration of absolute ethanol (1 mL/animal). Animals were euthanized 30 min after ethanol administration with sodium thiopental overdose (100 mg/kg, i.p.) and the stomachs were removed, opened along the lesser curvature, washed with normal saline, and examined in a blinded manner. The quantification of the ulceration induced by ethanol was performed using the Image*J*-NIH^*R*^ computer program (National Institutes of Health, Washington D.C.) to calculate the ulceration index expressed as the percentage of ulcerated area in relation to the area of the stomach body.

### 2.5. Gastric Ulcer Induced by Indomethacin in Rats

Male Wistar rats were randomly distributed in five groups of 7 animals maintained in a solid fast of 18 h and treated orally with vehicle (0.1% Tween 80, 5 mL/kg), menthofuran (12.5, 25, 50, and 100 mg/kg), and ranitidine (60 mg/kg) 30 min before and 3 h after induction of gastric ulcers by administration of indomethacin (30 mg/kg, sc). The animals were kept in separate cages and, 6 h later, were killed; their stomachs were removed and opened along the lesser curvature to determine the number and area of ulceration and to calculate an ulcer index [24], using the computer program NIH-Image*J* (National Institutes of Health, Washington D.C.).

### 2.6. Gastric Ulcer Induced by Ischemia and Reperfusion in Rats

Male Wistar rats, divided into four groups with 7 animals, were treated with vehicle (0.1% Tween 80, 5 mL/kg), menthofuran (50 and 100 mg/kg), or N-acetylcysteine (NAC, 100 mg/kg), and 15 minutes later, they were anesthetized by association of ketamine (50 mg/kg) and xylazine (5 mg/kg) intramuscularly. Then, each animal was subjected to an approximately 3 cm incision in the abdominal wall of the left side of the body for location of the abdominal aorta artery and celiac artery, which was clamped for 30 minutes using a microvascular clamp. Elapsing 30 minutes of ischemia, the clamp was removed to allow reperfusion of the gastric mucosa for 60 minutes [25]. Subsequently, the animals were euthanized, the stomachs removed and opened along the lesser curvature, washed with saline, and mounted between glass plates. Quantitation of induced ulceration was performed using the computer program NIH-Image*J* (National Institutes of Health, Washington D.C.) for calculating the area of ulcerative lesion, expressed as percentage of ulcerated area in relation to the area of the stomach body.

### 2.7. Determination of Gastric Secretion in Pylorus Ligated Rats

The volume, pH, and titratable acidity of gastric secretion were determined in groups of male Wistar rats randomly distributed in four groups of 7 animals which underwent ligature of the pylorus, according to Shay et al. [26]. Each animal was anesthetized by association of ketamine (50 mg/kg) and xylazine (5 mg/kg) intramuscularly and a longitudinal incision of about 2 cm was made in the abdominal wall, the stomach was located, and the pyloric sphincter was ligated with suture line, as well as the abdominal wall. Then, the animals were intraduodenally treated with vehicle (0.1% Tween 80, 5 mL/kg), menthofuran (50 and 100 mg/kg), or carbenoxolone (250 mg/kg) and, after 4 h, were sacrificed. The stomachs were removed to collect the gastric juice, and additionally, glandular segments from the stomachs were excised for determination of gastric wall mucus content and catalase activity, as described below.

### 2.8. Determination of the Gastric Wall Mucus Content in Pylorus Ligated Rats

Male Wistar rats randomly distributed in four groups of 7 animals underwent ligature of the pylorus, according to Shay et al. [26]. The animals were intraduodenally treated with vehicle (0.1% Tween 80, 5 mL/kg), menthofuran (50 and 100 mg/kg), or carbenoxolone (250 mg/kg) and, after 4 h, were sacrificed. A body stomach fragment from each animal subjected to pylorus ligation protocol was removed, weighed, and immediately transferred to 7 mL Alcian blue solution (0.25% weight/volume in sodium acetate buffer 0.05 M, pH 5.80 with 0.16 M sucrose) and incubated for 2 h at room temperature. Free dye was removed by successive washes for 15 and 45 minutes with 0.25 M sucrose solution. The dye attached to the gastric wall mucus was extracted by immersion in 5 mL of 0.5 M MgCl_2_ solution for 2 h, with 1 min agitation every 30 min. A sample of 4 mL of the dye bound to mucus was vigorously stirred with an equal volume of diethyl ether, and the resulting emulsion was centrifuged at 3000 rpm for 10 min. The optical density of Alcian blue in the aqueous layer was read in UV-VIS (Biospectro SP-220, EQUIP Ltda., Curitiba, Brazil) at 598 nm using distilled water as a blank. The result was expressed as *μ*g per weight (g) of the body stomach wall.

### 2.9. Determination of the Gastric Wall Catalase Activity of Pylorus Ligated Rats

The catalase activity in the gastric mucosa of pylorus ligated male rats was determined by the method of Beers and Sizer [27] in fragments of the wall of the stomach of 7 animals/group intraduodenally treated with vehicle (0.1% Tween 80, 5 mL/kg), menthofuran (50 and 100 mg/kg), or carbenoxolone (250 mg/kg). The fragments were weighed and homogenized in potassium phosphate buffer 0.05 M, pH 7.4, and centrifuged at 3,000 rpm for 15 min. A buffered solution of 0.059 M hydrogen peroxide was used as substrate solution to the test. The enzyme activity was determined by reading the absorbance decrease at 240 nm of the substrate between the first- and sixth-minute reactions. The results were expressed as the velocity of degradation of hydrogen peroxide in mM/min·g body stomach wall.

### 2.10. Determination of the Gastric Wall Nonprotein Sulfhydryl (NPSH) Group Content in Ethanol-Induced Gastric Ulcer Rats

Male Wistar rats, divided into four groups with 7 animals, were maintained on solid fast for 18 h and treated orally with distilled water (sham-control group, 5 mL/kg), 0.1% Tween-80 (vehicle group; 5 mL/kg), menthofuran (50 mg/kg), or N-acetylcysteine (200 mg/kg). One hour later, gastric lesions were induced by oral administration of absolute ethanol (5 mL/kg), except the control group (treated with distilled water 5 mL/kg), and after 30 minutes, the animals were sacrificed; their stomachs removed, washed, and opened along the lesser curvature. The concentration of nonprotein sulfhydryl groups (NPSH) was determined according to the method of Sedlak and Lindsay [28] in fragments (100 mg) of glandular stomach that were homogenized in 1 mL of 0.02 M EDTA. Aliquots of 400 *μ*L were mixed with 400 *μ*L 20% trichloroacetic acid, stirred, and centrifuged (3,000 rpm, 15 min). Then, 400 *μ*L of the supernatant was added to 800 *μ*L of 0.4 M Tris buffer pH 8.9 and 20 *μ*L of dithio-bis-nitrobenzoic acid (Ellman's reagent) 0.01 M. Absorbance at 412 nm was used to calculate the NPSH concentration expressed as *μ*g/g of tissue.

### 2.11. Gastric Wall Malondialdehyde (MDA) Content of Ethanol-Induced Gastric Ulcer Rats

Male Wistar rats (7 animals/group), previously submitted to the protocol of absolute ethanol and orally treated with distilled water (Sham-control group, 5 mL/kg), 0.1% Tween-80 (vehicle group; 5 mL/kg), menthofuran (50 mg/kg), or N-acetylcysteine (200 mg/kg), were used to evaluate the MDA concentrations in gastric wall fragments (100 mg) homogenized in cold 1.15% KCl solution, using the method of Uchiyama and Mihara [29]. Briefly, 250 *μ*L of homogenate were added to 1.5 mL of 1% H_3_PO_4_ and 0.5 mL of 0.6% thiobarbituric acid. Then, the mixture was stirred, heated in boiling water bath for 45 min, and immediately cooled in an ice water bath. Next 4 mL of *n*-butanol was added, and after stirring for 1 min, the butanol layer was separated by centrifugation at 1,200 g for 10 min. The absorbance difference at 535 and 520 nm was used to calculate the MDA concentration, expressed as *μ*g/g tissue.

### 2.12. Gastric Wall Myeloperoxidase Activity (MPO) of Ethanol-Induced Gastric Ulcer Rats

Male Wistar rats (7 animals/group), previously submitted to the protocol of absolute ethanol and orally treated with distilled water (Sham-control group, 5 mL/kg), 0.1% Tween-80 (vehicle group; 5 mL/kg), menthofuran (50 mg/kg), or N-acetylcysteine (200 mg/kg), were used to evaluate the myeloperoxidase activity (MPO) in gastric wall fragments (100 mg) homogenized in 1 ml of potassium phosphate buffer 0.1 M pH 7 with 0.5% hexadecyltrimethylammonium (HTAB), using the method of Bradley et al. [30]. After homogenization, the material was centrifuged (4,000 rpm, 7 min, 4°C) and the pellet was resuspended. The MPO activity was determined by measuring the change in absorbance at 450 nm using o-dianisidine dihydrochloride and 1% hydrogen peroxide as color reagents. The results were plotted as MPO units/mg tissue.

### 2.13. Statistical Analysis

The results were expressed as the mean ± standard error of the mean (S.E.M) and comparisons between the groups were tested by one-way ANOVA and Tukey's post hoc test. The differences between the groups were regarded as significant at *p* < 0.05. The software used was the GraphPad Prism, version 5.0.

## 3. Results

### 3.1. Acute Oral Toxicity Evaluation

The results revealed no signs of systemic toxicity following administration of menthofuran at a dose of 2,000 mg/kg (v.o). There were no behavioral changes and no deaths during the observation time of 14 days.

### 3.2. Effect of Acute Gastric Ulcer Induced by Ethanol in Rats

**Figure 1:**
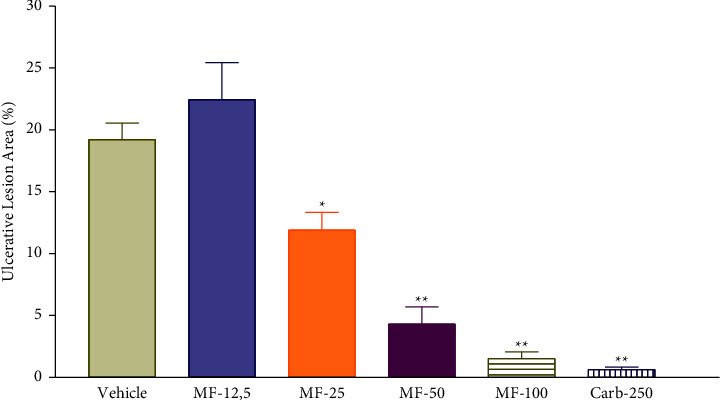
Ulcerative lesion area in rats after treatment with vehicle (1% Tween-80, 5 mL/kg), menthofuran (12.5, 25, 50, and 100 mg/kg), and carbenoxolone (250 mg/kg) in an experimental model of gastric ulcers induced by ethanol. The data represent the mean ± S.E.M. of 7 animals/groups. ^*∗*^*p* < 0.05 and ^*∗∗*^*p* < 0.01 compared to vehicle group (one-way ANOVA followed by Tukey test).

Menthofuran showed gastroprotective activity against gastric ulcer induced by absolute ethanol (Figure 1), significantly reducing the area of ulcerative lesions at doses of 25 (11.90 ± 1.43%), 50 (4.30 ± 1.40%), and 100 mg/kg (1.51 ± 0.55%) compared to the vehicle group (19.23 ± 1.33%), the latter similarly to the effect of carbenoxolone 250 mg/kg (0.62 ± 0.20%). At a dose of 12.5 mg/kg, menthofuran did not promote reduction in ulcerative lesion area (22.44 ± 2.99%).

### 3.3. Effect of Menthofuran on Gastric Ulcer Induced by Indomethacin

**Figure 2:**
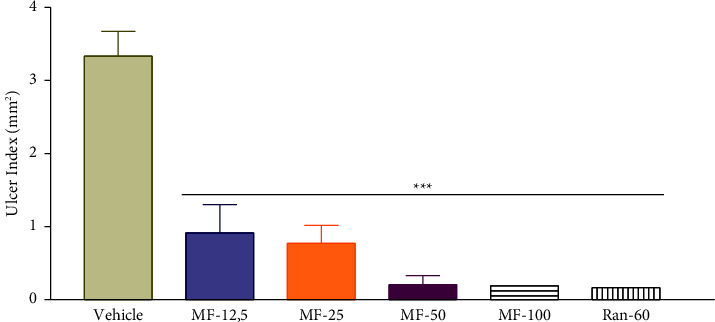
Ulcer index in rats after treatment with vehicle (1% Tween-80, 5 mL/kg), menthofuran (12.5, 25, 50, and 100 mg/kg), and ranitidine (60 mg/kg) in an experimental model of gastric ulcers induced by indomethacin. The data represent the mean ± S.E.M. of 7 animals/groups. ^*∗∗∗*^*p* < 0.001 compared to the vehicle group (one-way ANOVA followed by Tukey test).

In indomethacin-induced ulcer model (Figure 2), menthofuran significantly decreased the ulcer index (mm^2^) in the groups treated with doses of 12.5 mg/kg (0.92 ± 0.38), 25 mg/kg (0.77 ± 0.24), 50 mg/kg (0.21 ± 0.1), and 100 mg/kg (0.19 ± 0.19), as well as ranitidine 60 mg/kg (0.17 ± 0.11), compared with the vehicle group (3.33 ± 0.34).

### 3.4. Effect of Menthofuran on Gastric Ulcer Induced by Ischemia and Reperfusion

**Figure 3:**
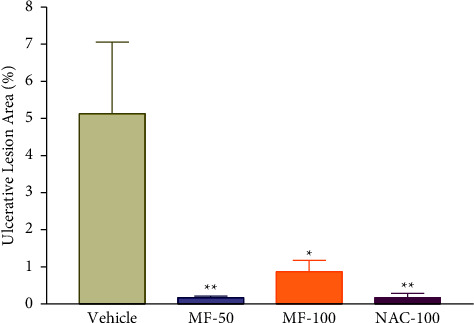
Ulcerative lesion area in rats after treatment with vehicle (1% Tween-80, 5 mL/kg), menthofuran (50 and 100 mg/kg), and N-acetylcysteine (NAC, 100 mg/kg) in an experimental model of gastric ulcers induced by ischemia and reperfusion. The data represent the mean ± S.E.M. of 7 animals/groups. ^*∗*^*p* < 0.05 and ^*∗∗*^*p* < 0.01 compared to the vehicle group (one-way ANOVA followed by Tukey test).

Menthofuran inhibited the appearance of gastric lesions induced by the occlusion of the celiac artery followed by reperfusion (Figure 3). The areas of ulcerative lesions in animals treated with the tested doses of 50 mg/kg (0.17 ± 0.04%) and 100 mg/kg (0.87 ± 0.30%) and with N-acetylcysteine 100 mg/kg (0.17 ± 0.11) were significantly lower compared with animals treated with vehicle (5.13 ± 1.93%).

### 3.5. Effect of Menthofuran on the Gastric Juice pH of Pylorus Ligated Rats

**Figure 4:**
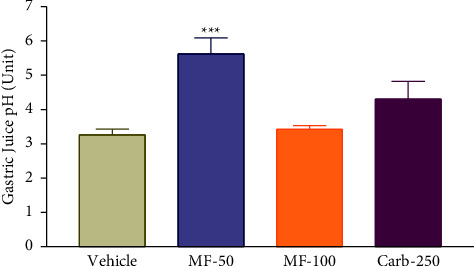
Gastric juice pH after intraduodenal treatment with vehicle (1% Tween-80, 5 mL/kg), menthofuran (50 and 100 mg/kg), and carbenoxolone (250 mg/kg) of pylorus ligated rats. The data represent the mean ± S.E.M. of 7 animals/groups. ^*∗∗∗*^*p* < 0.01 compared to the vehicle group (one-way ANOVA followed by Tukey test).

The effect of menthofuran on the gastric juice pH is shown in Figure 4. Menthofuran significantly increased the pH of gastric juice at a dose of 50 mg/kg (5.62 ± 0.47) but did not cause any change in the dose of 100 mg/kg (3.42 ± 0.11), as well as carbenoxolone 250 mg/kg (4.31 ± 0.51) compared to the vehicle (3.26 ± 0.16).

### 3.6. Effect of Menthofuran on the Gastric Juice Volume of Pylorus Ligated Rats

**Figure 5:**
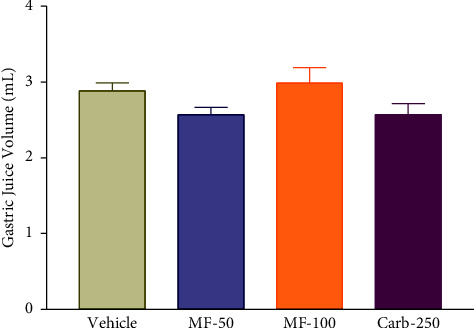
Gastric juice volume after intraduodenal treatment with vehicle (1% Tween-80, 5 mL/kg), menthofuran (50 and 100 mg/kg), and carbenoxolone (250 mg/kg) of pylorus ligated rats. The data represent the mean ± S.E.M. of 7 animals/groups.

There was no significant change in gastric juice volume (Figure 5) in animals treated with menthofuran at the evaluated doses of 50 mg/kg (2.6 ± 0.94) and 100 mg/kg (3.0 ± 0.20), or with carbenoxolone 250 mg/kg (2.6 ± 0.14) compared to vehicle (2.9 ± 0.10).

### 3.7. Effect of Menthofuran on the Gastric Juice Acidity of Pylorus Ligated Rats

**Figure 6:**
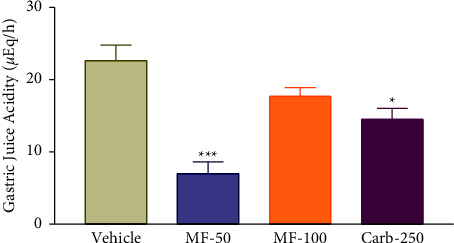
Gastric juice acidity after intraduodenal treatment with vehicle (1% Tween-80, 5 mL/kg), menthofuran (50 and 100 mg/kg), and carbenoxolone (250 mg/kg) of pylorus ligated rats. The data represent the mean ± S.E.M. of 7 animals/groups. ^*∗*^*p* < 0.05 and ^*∗∗∗*^*p* < 0.01 compared to the vehicle group (one-way ANOVA followed by Tukey test).

The intraduodenal administration of 50 mg/kg of menthofuran provoked a significant (*p* < 0.05) reduction in the total acidity of (6.98 ± 1.61). Carbenoxolone significantly reduced the gastric juice acidity (14.51 ± 1.53). However, menthofuran at a dose of 100 mg/kg showed no effect on this parameter (17.68 ± 1.20), compared with the vehicle (22.64 ± 0.16) (Figure 6).

### 3.8. Effect of Menthofuran on the Gastric Wall Mucus Content of Pylorus Ligated Rats

**Figure 7:**
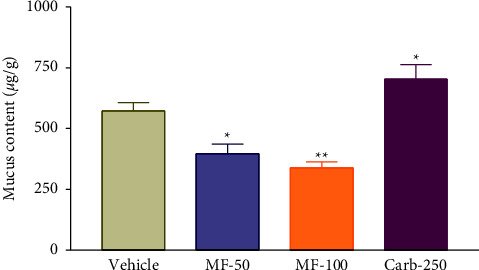
Gastric wall mucus content after intraduodenal treatment with vehicle (1% Tween-80, 5 mL/kg), menthofuran (50 and 100 mg/kg), and carbenoxolone (250 mg/kg) of pylorus ligated rats. The data represent the mean ± S.E.M. of 7 animals/groups. ^*∗*^*p* < 0.05 and ^*∗∗*^*p* < 0.01 compared to the vehicle group (one-way ANOVA followed by Tukey test).

The effects of menthofuran on changes in the amounts of gastric wall mucus in four-hourpylorus-ligated rats are depicted in Figure 7. There was a significant decrease in mucus content adhered to the gastric wall at the doses of 50 mg/kg (397.11 ± 32.94) and 100 mg/kg (338.15 ± 20.60), but the mucus content was increased in the presence of carbenoxolone (727.53 ± 44.58) compared to the vehicle group (573.06 ± 28.32).

### 3.9. Effect of Menthofuran on the Gastric Wall Catalase Activity of Pylorus Ligated Rats

**Figure 8:**
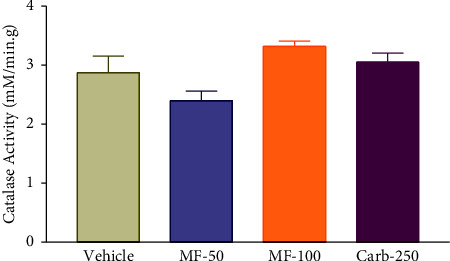
Gastric wall catalase activity after intraduodenal treatment with vehicle (1% Tween-80, 5 mL/kg), menthofuran (50 and 100 mg/kg), and carbenoxolone (250 mg/kg) of pylorus ligated rats. The data represent the mean ± S.E.M. of 7 animals/groups.

There was no significant difference in catalase activity between the groups treated with menthofuran 50 mg/kg (2.40 ± 0.16) and 100 mg/kg (3.32 ± 0.09), or with carbenoxolone 250 mg/kg (3.05 ± 0.15) compared to the vehicle group (2.87 ± 0.28) (Figure 8).

### 3.10. Effect of Menthofuran on the Gastric Wall Nonprotein Sulfhydryl (NPSH) Group Content of Ethanol-Induced Gastric Ulcer Rats

**Figure 9:**
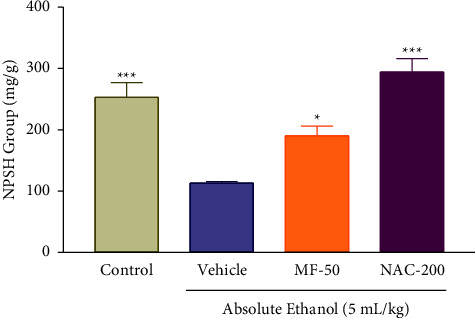
Gastric wall nonprotein sulfhydryl (NPSH) group content after oral treatment with vehicle (1% Tween-80, 5 mL/kg), menthofuran (MF, 50 mg/kg), and N-acetylcysteine (NAC, 200 mg/kg) of ethanol-induced gastric ulcer rats. The data represent the mean ± S.E.M. of 7 animals/groups. ^*∗∗*^*p* < 0.01 and ^*∗∗∗*^*p* < 0.001 compared to the vehicle group, (one-way ANOVA followed by Tukey test).

In the animals subjected to ethanol-induced gastric ulcers, the content of NPSH decreased significantly in the vehicle group (113.05 ± 4.77) compared to the control group (253.06 ± 23.76), and they were significantly restored in the groups treated with menthofuran at a dose of 50 mg/kg (190.62 ± 15.78) and with N-acetylcysteine 200 mg/kg (294.16 ± 19.65) compared with the vehicle group (Figure 9).

### 3.11. Effect of Menthofuran on the Gastric Wall Malondialdehyde (MDA) Content of Ethanol-Induced Gastric Ulcer Rats

**Figure 10:**
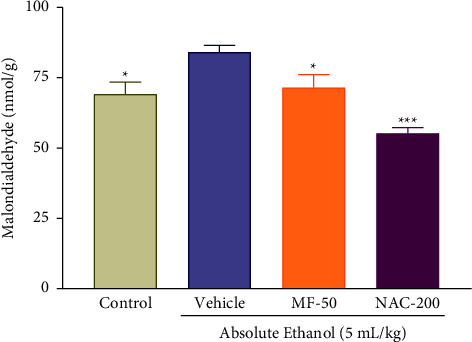
Gastric wall malondialdehyde (MDA) content after oral treatment with vehicle (1% Tween-80, 5 mL/kg), menthofuran (MF, 50 mg/kg), and N-acetylcysteine (NAC, 200 mg/kg) of ethanol-induced gastric ulcer rats. The data represent the mean ± S.E.M. of 7 animals/groups. ^*∗*^*p* < 0.05 and ^*∗∗∗*^*p* < 0.001 compared to the vehicle group (one-way ANOVA followed by Tukey test).

MDA levels increased significantly in animals subjected to ethanol-induced gastric ulcers (vehicle group, 89.49 ± 5.49 nmol/g) compared to the control group (69.35 ± 4.29 nmol/g), and they were significantly reduced in the groups treated with menthofuran at a dose of 50 mg/kg (71.70 ± 4.70 nmol/g) and N-acetylcysteine 200 mg/kg (51.36 ± 4.07 nmol/g) compared with the vehicle group (Figure 10).

### 3.12. Effect of Menthofuran on the Gastric Wall Myeloperoxidase Activity (MPO) of Ethanol-Induced Gastric Ulcer Rats

**Figure 11:**
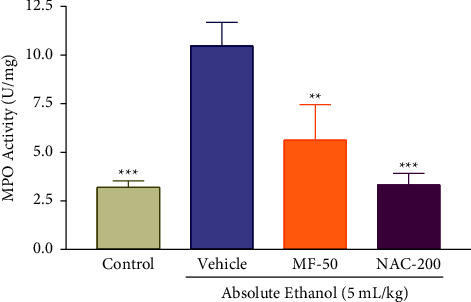
Gastric wall myeloperoxidase activity after oral treatment with vehicle (1% Tween-80, 5 mL/kg), menthofuran (MF, 50 mg/kg), and N-acetylcysteine (NAC, 200 mg/kg) of ethanol-induced gastric ulcer rats. The data represent the mean ± S.E.M. of 7 animals/groups. ^*∗*^*p* < 0.05 and ^*∗∗∗*^*p* < 0.001 compared to the vehicle group (one-way ANOVA followed by Tukey test).

MPO levels were significantly increased in the vehicle group (10.47 ± 1.21) compared to the control group (3.19 ± 0.33) and were significantly reduced by treatment with menthofuran 50 mg/kg (5.62 ± 1.82), and N-acetylcysteine 200 mg/kg (3.32 ± 0.59) compared to the vehicle (Figure 11).

## 4. Discussion

This study investigated the gastroprotective effect of menthofuran in animal models of gastric ulcers. For this, acute toxicity was initially assessed, and in the second time, it has been determined the antiulcerogenic effect of this monoterpene and its influence on gastric secretion to identify the possible mechanisms involved in this activity. The findings of the research show that the gastroprotective activity of menthofuran has similarity to other monoterpenes and no toxicity was observed. The results showed that the menthofuran, administered orally, has antiulcer activity against gastric ulcers induced by ethanol, indomethacin, and ischemia followed by reperfusion in rats. Additionally, in gastric ulcers induced by ethanol, menthofuran completely restored MDA levels and MPO activity, and partly NPSH, of the rat gastric wall. Furthermore, the menthofuran intraduodenally administered at a dose of 50 mg/kg, but not at 100 mg/kg, increased the pH and decreased the acidity of gastric secretion in rats subjected to pylorus ligation. However, by using this same model study, the menthofuran caused reduction of mucus content adhered to the gastric wall and did not alter the activity of catalase.

In assessing the acute toxicity of menthofuran, there were nonobserved toxic effects on the respiratory system, musculoskeletal system, and nervous system, whereas 100% survival of the animals treated with the dose of 2000 mg/kg was not possible to determine the median lethal dose (LD50). After this step, it initiated the protocols to assess the gastroprotective activity.

Gastric lesions are among the main diseases of the gastrointestinal tract, and its pathogenesis is related to a complex multifactorial process resulting from an imbalance between the protective and aggressive factors on the gastric mucosa [31, 32].

The menthofuran reduced gastric ulcer caused by ethanol showing that it may interfere with one or more mechanisms activated by ethanol in the induction of ulcers on the gastric mucosa. Ethanol-induced gastric ulcer has been widely used for the evaluation of antiulcerogenic activity of natural products [32–36]. In this model of gastric damage induction, ethanol produces extensive haemorrhagic and necrotic areas on the gastric mucosa of animals [4]. Ethanol induces ulcers by alteration of cellular homeostasis and it reduces gastric mucosal blood flow and mucus and bicarbonate production in the gastric lumen. This substance decreases endogenous glutathione and prostaglandin levels and increases ischemia, gastric vascular permeability, acid “back diffusion,” histamine release, efflux of sodium and potassium, influx of calcium, generation of free radicals, and the production of leukotrienes [4, 37]. Ethanol is also capable of promoting increased lipid peroxidation, increased release of endothelin, and degranulation of mast cells, predominantly in the glandular portion of the stomach [38].

Prostaglandins and nitric oxide (NO) are important factors involved in gastric defense mechanisms through the regulation of mucus secretion, acid and alkaline secretion, epithelial fluid, and mucosal blood flow [24, 39]. To assess the involvement of the prostaglandins in the effect of menthofuran, it was used the model of gastric ulcers induced by indomethacin, one nonsteroidal-anti-inflammatory drug (NSAID) that reduces prostaglandins levels [5].

NSAIDs are drugs routinely used throughout the world; however, they are known to induce gastric mucosal damage including gastritis, bleeding, ulceration, and perforation in humans and experimental animals [40]. These effects occur through the inhibition of the cyclooxygenase (COX) enzymes, resulting in a marked decrease in the levels of prostaglandins [41], but the reactive-oxygen-species (ROS)-induced enhancement in lipid peroxidation plays an important role in the mechanism of gastric damage induced and the increase in free radical metabolites depends possibly upon neutrophil activation and is associated with the significant increase in lipid peroxidation, the fall in the gastric blood flow at ulcer margin, and the excessive release of the proinflammatory cytokine such as IL-1b [40].

Menthofuran showed a mucosal gastroprotective effect suggesting a possible involvement of prostaglandins. Antiulcerogenic action against lesions induced by indomethacin was detected in study with menthol (50 mg/kg), a monoterpene similar to menthofuran that displayed a gastroprotective activity through antiapoptotic, antioxidant, and anti-inflammatory mechanisms [42].

In another study, (-)-Carveol (p-Mentha-6,8-dien-2-ol), a monoterpene found in species such as *Cymbopogon giganteus* and *Illicium pachyphyllum* and in spices such as*Carum carvi* (cumin), has an important gastroprotective activity involving multiple mechanisms of action, such as cytoprotective mechanisms (participation of sulfhydryl groups, nitric oxide, KATP channels, mucus secretion, and prostaglandins), antisecretory (reduction in the volume of gastric secretion), antioxidants (increase in GSH and SOD with reduction in MDA and MPO), and immunoregulatory (reduction in TNF-*α* and IL-1*β* and increase in IL-10) [43].

Pylorus ligature is an experimental procedure used in studies of antiulcer drugs that could decrease the acid output and increase the amount of gastric mucus secretion [26]. In this study, the menthofuran at doses of 50 and 100 mg/kg did not alter the mean values of the gastric juice volume; however, at a dose of 50 mg/kg, it increased pH and decreased acidity when compared to the vehicle. These results suggest that the mucosal protective mechanism of menthofuran is mainly related to the reduction of acidic secretion. The mechanism that increases the acidity of the stomach involves the interaction of histamine, gastrin, and acetylcholine with its receptors on parietal cells, triggering the pump protons that are responsible for the secretion of H^+^ ion in the gastric lumen [44]. However, it is not possible to determine which of this pathway could be activated by menthofuran.

Menthofuran reduced the mucus adhered to gastric wall in the pylorus ligated model. Mucus is an important factor in mucosal protection of the gastrointestinal tract because it constitutes a semipermeable barrier coating [45]. As the used protocol did not quantify the content of free mucus, this constitutes a limitation of this study in respect to the effect of menthofuran in tee total mucus of the gastric wall.

Blood flow is an important factor in the protection of normal gastric mucosa and in the protection and healing of damaged mucosa by supplying the mucosa with oxygen and HCO_3_^−^ and by removing H^+^ and toxic agents diffusing from the lumen into the mucosa [46]. Ischaemia and reperfusion are known to induce gastric lesions, predominantly due to excessive formation of reactive oxygen metabolites, adhesion of neutrophils to endothelial cells, microvascular dysfunction, gastric acid secretion, endogenous histamine, and gastrin release [47].

In the ulcer model induced by ischemia and reperfusion, the monoterpene menthofuran reduced the lesion area and its effect was similar to that obtained with the standard drug, N-acetylcysteine (NAC), a substance that is a potent scavenger free radical [48]. The fact that menthofuran presents similar effect of NAC may indicate that it has antioxidant activity that protected the gastric mucosa against lesions.

This result corroborates the study by Oliveira et al. [49] that when performing this protocol, it was found that carvacrol, also a monoterpene, reduced lesion area caused by ischemia and reperfusion. Among the main mechanisms involved in the gastroprotective activity of carvacrol in that study, there were increased cytoprotective as mucus and prostaglandin, opening of potassium channels, restoring antioxidant levels as NPSH, and catalase.

The imbalance between the oxidant and antioxidant systems leads to a biological condition defined as oxidative stress that contributes to the formation of reactive oxygen species, which are unstable molecules resulting from various processes in the body that can be highly harmful and cause a series of diseases [50]. Oxidative stress is closely related to the pathogenesis of various diseases, and it is postulated that it plays a crucial role in the induction of gastric mucosal lesions induced by ethanol. ROS promotes oxidative stress, causing deleterious effects and cell death, leading to degenerative diseases, cancer, ulcers, and atherosclerosis [44, 51, 52].

Several plants, plant extracts, and phytochemicals with antioxidant activity, such as many phenolic compounds, flavonoids, and terpenoids, play an important role in preventing oxidative damage, whereas these agents neutralize and break the free radical chains and can contribute to control the oxidative stress-initiated disease [51, 53]; they have potential utility as pharmacotherapeutic intervention to scavenge ROS and ameliorate effects against mutagens, carcinomas, and inflammatory pathological processes [51, 57, 58].

In this context, the isolated bioactive furan-type derivative compounds isolated from *Persicaria hydropiper* L. exhibited considerable activity against free radicals and target enzymes implicated in type 2 diabetes mellitus [53]. Furthermore, the antioxidant properties of the phytocompounds, such as menthol, a-terpinene, 1-p-menthen-9-alc, lilac aldehyde, limonene diol, and others, that seem to contribute profoundly to antidiabetic effects of honey by mitigating the oxidative stress and protecting against tissue damage [56].

In this study, it has become relevant to assess the action of the menthofuran on the effect produced by the ROS ethanol-induced acute ulcer. Our results demonstrate that ethanol promoted the depletion of mucosal NPSH. The administration of NAC (200 mg/kg, po) was effective in restoring NPSH gastric levels. Similarly, menthofuran (50 mg/kg, po) was also able to restore; the NPSH levels in gastric mucosa reduced after administration of absolute ethanol. Therefore, it is suggested that one possible mechanism responsible for the gastroprotective action of this monoterpene in the ethanol-induced lesions is the maintenance of high levels of NPSH in the gastric mucosa. These results are in line with the study of Santos et al. [57] who found the reestablishment of GSH after treatment with the monoterpene Cineol in the gastric injury model induced by ethanol using a dosage model [58] similar to that performed in this study.

Catalase (CAT) is a common enzyme found in nearly all living organisms, which has antioxidant action related to the maintenance of the gastric integrity, since it acts by accelerating the breakdown of H_2_O_2_ into water and oxygen [52]. In this study, treatment with menthofuran produced no change in CAT activity, which may indicate that the antioxidant activity of this substance does not involve the path of catalase.

To evaluate the effect of menthofuran on lipid peroxidation promoted by gastric damage induced by ethanol beams of quantitation of malondialdehyde (MDA), this organic compound is derived from the degradation of polyunsaturated lipids and is synthesized during lipid peroxidation. MDA is thus a widely used marker in the measurement of oxidative damage resulting from interaction between free radicals and lipid [59]. Menthofuran decreased the MDA levels in ethanol-induced gastric ulcers, suggesting a possible involvement of this monoterpene via antioxidant, reducing the levels of lipid peroxidation. In a study conducted before further strengthening this hypothesis, the monoterpene thymoquinone also reduced MDA levels after the gastric ulcer model induced by ethanol [34].

The determination of MPO activity was used as a parameter to evaluate the inflammatory response mediated by neutrophils, since it is a proteolytic enzyme present in cytoplasmatic granules of the polymorphonuclear neutrophils and participates in innate immune defense mechanisms through the formation of ROS, and when released, the MPO can react with the hydrogen peroxide formed by NADPH oxidase and increase the toxic potential of the oxidant [60, 61]. Therefore, the lower activity of MPO found in the group treated with menthofuran in gastric ulcer ethanol-induced indicates reduced neutrophilic infiltrate and points to a relevant gastric anti-inflammatory activity, which appears to contribute significantly to the gastroprotective effect promoted by this monoterpene.

## 5. Conclusion and Perspectives

The present study demonstrates that menthofuran presented gastroprotective effects against gastric mucosal damage induced by ethanol, indomethacin, and ischemia and reperfusion, which appear to be mediated, at least in part, by activating the NOS pathway, endogenous prostaglandins, reduced gastric juice acidity, increased concentration of NPSH groups, and reduced lipidic peroxidation. The possible involvement of the antioxidant system in the action of this monoterpene was evidenced by the increased concentration of NPSH and the decrease of lipid peroxidation, which is demonstrated by the reduction of MDA and MPO. The reduction in the latter biomarker also suggests anti-inflammatory activity in the gastric wall. These findings indicate the therapeutic potential of menthofuran to be used as an effective gastroprotective agent.

However, the current literature available is limited; the authors emphasize the need for more detailed *in vitro* and *in vivo* studies to assess the exact mechanism involved in the gastroprotective action of menthofuran in order to provide additional data for understanding the molecular and signaling mechanisms and that can generate consistent evidence for conducting clinical trials to assess the quality, safety, and therapeutic efficacy of this natural product.

## Data Availability

Data are available upon request.
